# Neurostructural correlate of math anxiety in the brain of children

**DOI:** 10.1038/s41398-018-0320-6

**Published:** 2018-12-10

**Authors:** Karin Kucian, Ursina McCaskey, Ruth O’Gorman Tuura, Michael von Aster

**Affiliations:** 1 Center for MR-Research, University Children’s Hospital, Zurich, Switzerland; 2 Children’s Research Center, University Children’s Hospital, Zurich, Switzerland; 3 Neuroscience Center Zurich, University of Zurich and ETH Zurich, Zurich, Switzerland; 4 Zurich Center for Integrative Human Physiology, University of Zurich, Zurich, Switzerland; 5 Clinic for Child and Adolescent Psychiatry, German Red Cross Hospitals, Berlin, Germany

## Abstract

Adequate mathematical competencies are currently indispensable in professional and social life. However, mathematics is often associated with stress and frustration and the confrontation with tasks that require mathematical knowledge triggers anxiety in many children. We examined if there is a relationship between math anxiety and changes in brain structure in children with and without developmental dyscalculia. Our findings showed that math anxiety is related to altered brain structure. In particular, the right amygdala volume was reduced in individuals with higher math anxiety. In conclusion, math anxiety not only hinders children in arithmetic development, but it is associated with altered brain structure in areas related to fear processing. This emphasizes the far-reaching outcome emotional factors in mathematical cognition can have and encourages educators and researchers alike to consider math anxiety to prevent detrimental long-term consequences on school achievement and quality of life, especially in children with developmental dyscalculia.

## Introduction

Math anxiety meets all the criteria of a specific phobia such as feelings of tension, stress, frustration and anxiety when manipulating numbers or solving mathematical problems during daily life or in school situations. However, math anxiety is not only associated with immediate negative emotional reactions but also has detrimental long-term consequences for career choice, employment, and professional success. A recent study has shown that the central component of financial literacy can be traced to numeracy and the emotional attitudes towards numbers like math anxiety^1^. Nevertheless, math anxiety has only recently attracted the interests of the research community and teachers, psychologists, and neuroscientists have just begun to uncover the far-reaching negative consequences of mathematical anxiety.

Although it is unclear to what extent, math anxiety causes mathematical difficulties or vice versa, there is conclusive proof that math anxiety interferes with mathematical performance, especially with tasks requiring working memory. The most prominent theory explains this relationship by suggesting that worrying intrusive thoughts involved in math anxiety consume attentional recourses of working memory, which are therefore less available for current numerical cognition (reviewed by ref. ^2^).

Although math anxiety seems to be related to other measures of anxiety, like general anxiety or test anxiety, it cannot be reduced purely to these more general measures. It has been proposed that math anxiety should be considered a specific phobia, since math-anxious individuals show both elevated cognitive and physiological arousal due to a stimulus-specific and situation-specific learned fear (reviewed by refs. ^2,3^). Moreover, the prevalence of math anxiety has been reported to be as high as 25% (reviewed by ref. ^3^) and anxiety and negative attitudes towards mathematics seem to be more common than towards other school subjects. Females tend to express more anxiety about mathematics, but reported gender differences are inconsistent and math anxiety in females is thought to develop only during adolescence (reviewed by ref. ^3^). In general, mathematics anxiety appears to increase with age during childhood, but can already be present in children in the first grade (reviewed by ref. ^3^). However, the possible causes of math anxiety remain unknown and the question of why children develop math anxiety has been widely discussed (reviewed by ref. ^2^). The development of math anxiety is probably not only due to the vulnerability of negative experiences with mathematics in school and daily life, but also likely involves a broader spectrum of factors including genetic^4^ as well as non-genetic inheritance^5–8^ influencing both anxiety and mathematical cognition. Non-genetic effects have been demonstrated for math anxious parents or teachers. For instance, when parents are more math anxious, their children learn less math and have more math anxiety, but only if math-anxious parents provided frequent help with math homework^6^. Another study has shown that female teachers’ math anxiety affects girls’ math achievement^8^. Hence, the interaction of affective and cognitive domains is supposed to be particularly important in mathematics. High levels of math anxiety might lead to negative attitudes and worse performance. On the other hand, it is also plausible that prior low levels of math achievement might promote the development of math anxiety and avoidance behavior towards mathematics, resulting in even lower math skills. Although the specific causes and effects are unclear, math anxiety seems to be particularly common in children with math learning disabilities (e.g.^9^).

Developmental dyscalculia (DD) is a common specific learning disorder of mathematical abilities associated with severe problems in various aspects of number processing and calculation that cannot be explained by mental retardation, inappropriate schooling or poor social environment (reviewed by refs. ^10,11^). Particularly noteworthy is that children with DD are reported to develop internalizing problems such as anxieties^12^, especially in relation to mathematical situations^9^. Therefore, children with DD are an interesting group in which to study math anxiety.

Despite the progress made in understanding how math anxiety relates to mathematical performance, only limited attention has been devoted to improving our understanding of the neurobiology of math anxiety (reviewed by ref. ^13^). What brain imaging studies have unraveled so far is that math anxious individuals activate brain circuits to a greater extent, which are associated with negative emotional possessing (amygdala, prefrontal cortices)^14–16^, the experience of pain (insula)^17^, inhibiting irrelevant information^18^, or conflict processing^19^. It is interesting to note that the emotional control network seems to be upregulated even prior to mathematical performance, indicating that the anticipation of an upcoming mathematical task leads to greater activity^17,20,21^. Math-anxious subjects also tend to show stronger activation in regions related to mathematical performance (parietal cortices, fusiform gyri, hippocampus)^14,15,22^, the coordination of task demands and motivational factors during calculation (caudate, nucleus accumbens, hippocampus)^20^, self-generated errors in numerical tasks (insula)^19^, or the inhibition of incorrect responses during calculation (frontal cortices)^23^. These findings demonstrate that math anxiety can be attributed to altered brain functions in math-related areas or common neurobiological pathways typically involved in several types of anxiety. Evidence regarding brain substrates of anxiety and stress lend support to the claims that our brain not only reacts with adapted (functional) activation due to detrimental experiences, but that adversity even leads to structural changes of the brain (reviewed by ref. ^24^). However, to the best of our knowledge, no study to date has investigated possible structural alterations due to math anxiety.

Taken together, many children develop specific anxieties when confronted with mathematics in school or daily life, which go hand-in-hand with altered brain function. Moreover, there is a growing body of evidence that stressful events can even affect brain structure (reviewed by ref. ^24^). Therefore, the aim of the present study was to examine the relationship of mathematical anxiety and grey matter brain volume in typically achieving children and children with DD who are particularly vulnerable to the development of math anxiety.

## Materials and methods

### Subjects

A total number of 63 children participated in the present study, of which 20 were excluded due to missing of behavioral data (*N* = 3), image artefacts in the structural brain scans (*N* = 6), both missing behavioral data and image artefacts (*N* = 3), or inaccurate segmentation of brain images (*N* = 8). This resulted in a group size of 43 children between 7.8–15.9 years of age, consisting of 23 children with DD and 20 gender-matched and handedness-matched control children (CC) showing age appropriate math performance. To determine whether a correlation coefficient differs from zero with a large effect size (*r* = 0.5) and with an alpha (two-tailed) = 0.05 (Type I error rate) and a beta = 0.200 (Type II error rate) a total sample size of *N* = 29 would have been required. Groups differed slightly in age, whereas DD children were a bit older than CC children (*p* < 0.05). All children underwent detailed neuropsychological testing and MRI-measurement. Due to the wide age-range, different age-appropriate behavioral tests had to be conducted to identify children with DD. Children with DD below the age of 12 years fulfilled all the diagnostic criteria for DD according to the Neuropsychological Test Battery for Number Processing and Calculation in Children for grades 1–4 (ZAREKI-R)^25^. Children with DD older than 12 years did all not achieve basic mathematical competencies of the fourth grade according to the Basic Diagnostic in Mathematics for grades 4–8 (BASIS-Math)^26^. None of the participants had neurological or psychiatric disorders, were on medication, or had any contraindications for MRI. Table 1 summarizes demographic and behavioral data of all participants.

**Table 1:** Behavioral data of children with developmental dyscalculia (DD) and control children (CC)

	Total	DD	CC	Statistics
				*p*-value
All children				
Subjects (*N*)	43	23	20	–
Age (years)				
Range	7.9–15.9	9.4–15.9	7.9–15.8	
Mean (SD)	11.9 (2.3)	12.5 (2.0)	11.2 (2.4)	**0.048**
Gender (male/female)	10/33	5/18	5/15	0.801^#^
Handedness (right/ambidextrous/left)	33/6/4	18/2/3	15/4/1	0.419^#^
Mathematical anxiety^a^ (Intensity score)	2.8 (2.1)	3.4 (1.7)	2.1 (2.3)	0.008*
Number line performance^b^ (%)	6.1 (2.6)	6.1 (2.7)	6.0 (2.5)	0.817*
Addition^c^ (%)	90.3 (9.6)	85.9 (10.6)	95.5 (4.6)	0.000*
Subtraction^c^ (%)	78.9 (20.9)	67.3 (21.9)	91.8 (9.4)	0.000*
Intelligence^d^ (IQ)	103.6 (9.5)	98.6 (7.2)	109.4 (8.5)	0.000*
Working memory^e^ (Total score)	5.4 (1.9)	5.0 (1.8)	5.9 (2.0)	0.158
Children < 12 years				
Subjects (*N*)	21	8	13	–
Mathematical performance^f^ (PR)	38.7 (31.1)	7.9 (11.0)	55.3 (24.9)	0.000*
Arithmetical fluency^g^ (t)	47.2 (8.2)	41.0 (6.4)	51.0 (7.0)	**0.004**
Reading^h^ (t)	48.4 (6.2)	44.9 (5.2)	50.5 (5.9)	0.020*
Children > 12 years				
Subjects (*N*)	22	15	7	–
Mathematical performance^f^ (Total score)	57.7 (14.4)	50.3 (11.0)	73.6 (3.6)	**0.000**
Magnitude comparison^i^ (t)	44.4 (7.6)	39.5 (4.6)	52.1 (3.8)	**0.000**
Reading children (PR)	2.1 (18.3)	22.1 (20.9)	19.1 (12.9)	0.971*

^#^Chi-square tests were used for nominal data input

*Mann–Whitney-*U* tests were used for non-normal distributed data. For all other statistical comparisons between groups, two-sample *t*-tests were performed for data with normal distribution

^a^Mathematical anxiety is based on the mean of the mathematical anxiety intensity of the MAI-questionnaire (0 = no anxiety, 10 = high anxiety)

^b^Number line performance of children younger than 12 years is based on the percentage error between indicated and correct location of an Arabic digit, the solution of an addition or subtraction problem, and estimated number of dots on a paper-and-pencil number line task 0–100 (*N* = 21, 8 DD and 13 CC). For children older than 12 number line performance is measured by a computerized number line task 0–100 on which 20 visually presented Arabic digits had to be located by mouse-click (*N* = 22, 15 DD and 7 CC)

^c^Percentage of correctly solved addition and subtraction problems, respectively, in the number line task

^d^Mean IQ of children younger than 12 years is based on the mean of the subtests similarities, block design, digit span, picture concepts, vocabulary, and arithmetic of the WISC-IV and of children older than 12 years is based on the mean of the subtests similarities, block design, digit span and matrix reasoning of the WISC-IV

^e^Working memory capacity is based on the number of correctly repeated blocks of the CORSI-Block Suppression test

^f^Mathematical performance of children younger than 12 years (*N* = 20, 7 DD and 13 CC) is based on the total reached percentile rank (PR) in the ZAREKI-R test battery. Mathematical performance of children older than 12 years (*N* = 22, 15 DD and 7 CC) is assessed by the total score of the BASIS-Math test battery (Maximum is 83 points, whereas scores below 67 indicate that basic mathematical competencies of the fourth grade are not achieved)

^g^Arithmetical fluency of children younger than 12 years is based on the mean of correctly solved addition, subtraction, multiplication, and division problems within 2 min each of the HRT test (*N* = 21, 8 DD and 13 CC)

^h^Reading skills of children younger than 12 years (*N* = 21, 8 DD and 13 CC) is based on the subtest reading of the BUEGA test battery (t-value) and of children older than 12 years (*N* = 21, 14 MD and 7 CC) on the mean percentile rank (PR) of correctly read words and pseudowords of the SLRT-II

^i^Magnitude comparison skills of children older than 12 years (*N* = 18, 11 DD and 7 CC) is based on the mean t-values of the subtest quantity of the KFT 4–8 + R test battery

Statistically significant values are marked bold

Parents gave written consent and children received a voucher for their participation. The study was approved by the local ethics committee based on guidelines from the World Medical Association’s Declaration of Helsinki^27^.

### Cognitive assessments

#### Mathematical anxiety

Mathematical anxiety was assessed by the Math-Anxiety-Interview for German speaking primary school children (MAI)^28^. The MAI combines two different types of questions while four math related situation are verbally and pictorially presented (1st on the eve of a math test, 2nd math homework, 3rd math class, and 4th everyday/shopping). The child is initially asked to rate its anxiety intensity concerning the presented situation by an anxiety thermometer from 0 to 10. In a second step the different components of anxiety (affective, cognitive, behavioral, and physiological) are explored. The child is asked to estimate, to what extend specific statements apply to the particular situation, e.g., “I cannot get a word out”.

For the present study we have chosen the mean of experienced math anxiety intensity of all four situations which provides a valid and reliable measure (shown by 28) from 0 = no anxiety to 10 = very strong math anxiety in primary school children.

#### Mathematical performance

Two different age-appropriate test batteries were used to assess numerical and mathematical performance in children younger or older than 12 years, respectively:

Numerical achievement in children younger than 12 years were assessed using the standardized Neuropsychological Test Battery for Number Processing and Calculation in Children (ZAREKI-R)^25^. This neuropsychological battery examines basic skills in calculation and arithmetic and aims to identify and characterize the profile of mathematical abilities in children with DD from the 1st to 4th grade level. It is composed of 11 subtests, such as reverse counting, subtraction, number reading, dictating, visual estimation of quantities, and digit span forward and backward. Criteria for DD were met if a child’s performance in the ZAREKI-R was below the 10th percentile on average in three subtests or in the total reached percentile rank.

Numerical abilities of children older than 12 years were assessed using the German test battery Basic Diagnosis in Mathematics Education for Grades 4–8 (BASIS-MATH 4–8)^26^. The test battery measures different numerical abilities at three difficulty levels such as counting, arithmetic, decimal system, text problems and part-whole-relationships. Criteria for DD are met if the performance falls under a total threshold score of 67 points (out of a total of 83 points), which indicates that mastery of basic mathematical concepts has not been reached.

#### Number line performance

The spatial representation of numbers was measured by a paper-and-pencil number line task in children younger than 12 years a computerized version for children older than 12 years:

Children below 12 years had to indicate with a pencil on a left-to-right oriented number line from 0 to 100 the location of 20 Arabic digits, results of 20 additions and 20 subtractions, or the estimated number of 10 different dot arrays. The error rate of the paper-and-pencil number line task was evaluated by measuring the distance in percent (% distance) relative to the position of the correct number for each trial. Mean percentage distance was then calculated over all trials (Arabic digits, additions, subtractions, dots), but only correctly calculated addition and subtraction problems were included. A detailed description of the task is described in a previous publication of our group^29^.

Children older than 12 years solved a computerized number line task 0–100 including 20 Arabic digits that had to be located on the number line by mouse-click^30^. Again, the mean distance between the correct and indicated location in percent was calculated.

#### Arithmetic

Addition and Subtraction skills were measured in all children by the percentage of correctly solved addition or subtraction problems that had to be calculated in the number line task described above^29,30^.

Arithmetic fluency was additionally evaluated in children younger than 12 years using the addition, subtraction, multiplication, and division subtests of the “Heidelberger Rechentest (HRT)”^31^. In this test, a list of 40 addition/subtraction/multiplication/division tasks is presented to the child and he/she is asked to solve as many problems as possible within 2 min. Hence, in contrast to the assessment of addition and subtraction skills in the number line task, the present test puts children under time pressure.

#### Magnitude comparison

Magnitude comparison skills of children older than 12 years was assessed by the subtest Quantity Comparison of the standardized Cognitive Abilities Test (KFT 4–8 + R)^32^. Adolescents had 10 min time to solve as many magnitude comparison problems as possible of increasing difficulty of totally 25 different trials. Always two magnitudes had to be compared and decided which is the larger or if both are equal. Trials include non-symbolic comparison of number of items (e.g., dots), non-symbolic and symbolic calculation problems, different unities (e.g., time, money, weight, and liters), surface areas, and fractions.

#### Intelligence

Estimated intelligence was measured by the mean of different subtest of the standardized test battery Wechsler Intelligence Scale for Children (WISC-IV)^33^. Mean IQ of children younger than 12 years is based on four verbal (vocabulary, arithmetic, similarities, and digit span) and two performance subtests (picture arrangement, block design) of the WISC-IV. Mean IQ of children older than 12 years was assessed by the mean of the subtests similarities, block design, digit span, and matrix reasoning of the WISC-IV.

#### Working memory

Spatial working memory was assessed by the Block Suppression Test^34^. This test is based on the CORSI-Block Tapping test^35^ and requires the subject to reproduce every second block in a given sequence of touched cubes on a wooden board as the examiner demonstrated. While the sequences gradually increase in length, the number of cubes last tapped in two consecutively correct sequences is defined as the maximum spatial working memory span.

#### Reading skills

Reading skills were measured by standardized age appropriate German reading tests. Reading in children younger than 12 years was assessed by the subtest reading of the test battery “Basisdiagnostik Umschriebener Entwicklungsstörungen im Grundschulalter” (BUEGA)^36^. The subtest reading consists of two word lists (list 1 = 32 short words; list 2 = 24 longer words) that children had to read loud while number of errors and time was measured and influenced the reached t-value.

Reading performance in children older than 12 years was estimated by the reading-task from the standardized Salzburg Reading and Orthography Test (SLRT-II)^37^, which assesses word and pseudoword reading fluency of a 1-minute-reading-task. Because of lacking test norms in grades 7 and 8, values were obtained by interpolating the norms from the test manual (grade 6) and from Kronschnabel et al.^38^ (grade 9).

#### Handedness

Handedness was determined by the Edinburgh Handedness Inventory^39^.

### Brain imaging data acquisition

MRI data were acquired on a 3T General Electric Discovery 750 Scanner (GE Medical Systems, USA) using an 8-channel head coil. Three-dimensional anatomical images of the entire brain were obtained parallel to the anterior–posterior commissure line with a T1-weighted structural image using a spoiled gradient echo sequence (3D SPGR, number of slices = 172, slice thickness = 1 mm, no interslice skip, matrix size = 256 × 256, field of view = 256 mm, FA = 8°, TE = 3 ms (*N* = 28 subjects) or 5 ms (*N* = 24 subjects), TR = 10 ms (*N* = 28 subjects) or 11 ms (*N* = 24 subjects), scan duratio*N* = 3 min 52 s).

### Statistical analyses of behavioral data

Behavioral data were analyzed by IBM SPSS Statistics 22 Version 2. The Kolmogrorov-Smirnov test was used to assess normal distribution and the Levene’s test was used to statistically compare the variance between groups. DD and CC group differences were calculated by two-sample *t*-tests for normally distributed data and by the Mann–Whitney-*U* tests for non-normal data. Nominal data input (gender, handedness) was compared between DD and CC by means of chi-square tests. Since DD and CC groups differed in age, additionally multivariate analyses of variance (MANOVA) was conducted including age as confounding factor to exclude group differences based on age differences. Correlation analyses were performed by means of Pearson correlations for normal distributed data and by Spearman-Rho correlations for non-normal data. Finally, gender differences were evaluated by two-sample *t*-tests. In general, if parameters were available for all children, statistical analyses was performed including all children and additionally including age as nuisance factor. If parameters were only available for children younger or older than 12 years due to different age-appropriate neuropsychological testing, separate statistical analyses were performed for both age groups.

### Statistical analyses of brain imaging data

Brain images were processed by the automated structural analyses software FreeSurfer (v5.3.0, http://surfer.nmr.mgh.harvard.edu) on a Linux operating system, which enabled us to parcellate the brains into 26 subcortical and 148 cortical grey matter volumes based upon existing brain atlases. Detailed anatomical description of subcortical areas can be found in Table S1 and of cortical regions in Table S2 (see supplementary materials).

Default analyses settings were used to run the standard automated reconstruction pipeline “recon-all”, which has been described in prior methodological publications^40–43^. Main steps of the workflow include skull-stripping^44^, affine registration with Talairach atlas^45^, signal intensity normalization^46^, voxel-based grey and white matter segmentation, volume calculation, inflation and registration to a spherical surface atlas, allowing parcellation and labelling of subcortical^42^ and cortical regions^47^.

The quality of segmentation into grey and white matter was visually inspected and corrected where necessary by the recommended instructions on the FreeSurfer Wiki webpage (https://surfer.nmr.mgh.harvard.edu/fswiki) using TKMEDIT, an integrated tool in FreeSurfer. In particular, manual correction was performed in three steps: First, checking Talairach transformation, second, grey matter corrections in the brain mask (brainmask.mgz), and third, white matter corrections in the white matter mask (wm.mgz). Accordingly, before starting the recon-all workflow, first Talairach alignment was adjusted. The second step included deletion of obvious non-grey matter classified as grey matter, e.g., from a bad skull-stripping, as well as, adding control points in the white matter where white matter was included into the grey matter mask. After these corrections, a part of the recon-all pipeline was rerun (autorecon2-cp). Third, obvious non-white matter voxels classified as white matter were deleted from the white matter mask (wm.mgz) and lateral ventricles were filled where the automatic pipeline did not fill them entirely. Thereafter, part of recon-all workflow was rerun (autorecon2-wm).

Subcortical and cortical volumes were computed of 26 subcortical areas (9 left, 9 right, 8 central (FreeSurfer output aseg.stats)) based upon the existence of an atlas containing probabilistic information on the location of structures described by Fischl et al^42^. and of 148 cortical regions (74 left (FreeSurfer output lh.aparc.a2009s.stats), 74 right (FreeSurfer output rh.aparc.a2009s.stats)) based on the Destrieux atlas^48^. Detailed information about subcortical volumes can be found in Table S1, and about cortical volumes in Table S2.

Statistical analyses was performed using IBM SPSS Statistics 22 Version 2. The Kolmogrorov-Smirnov test was applied to assess normal distribution. For normal distributed data, partial correlation between intensity of math anxiety and left or right subcortical volumes, central volumes, or left or right cortical volumes was performed. Effects of total intracranial volume, addition and subtraction performance were controlled since math anxiety correlated significantly with addition and subtraction (see Table 2) and to control for general effects of total intracranial volume. Post-hoc correction for multiple comparisons was performed by false-discovery-rate (FDR). Since FDR correction assumes independency between factors, FDR correction was applied separately for left and right hemispheres. For non-normal data, additionally to the partial correlation a bootstrap estimation approach with 1000 samples and bias corrected and accelerated method as confidence interval was applied.

**Table 2:** Correlation between mathematical anxiety and behavioral data

	Mathematical anxiety		
	*N*	*r* or *r*_s_	*p*
All children			
Age	43	*r* = −0.112	0.475
Number line performance^a^	43	*r*_s_ = 0.072	0.645*
Addition^b^	43	*r*_s_ = −0.295	**0.055** *
Subtraction^b^	42	*r*_s_ = −0.360	**0.019** *
IQ^c^	43	*r* = −0.142	0.365
Working memory^d^	42	*r*_s_ = −0.102	0.519*
Children < 12 years			
Mathematical performance^e^	20	*r*_s_ = −0.178	0.454^*^
Arithmetical fluency^f^	21	*r* = −0.540	**0.011**
Reading^g^	21	*r* = −0.395	0.077
Children > 12 years			
Mathematical performance^e^	22	*r* = −0.176	0.432
Magnitude comparison^h^	18	*r* = −0.121	0.633
Reading^g^	21	*r*_s_ = 0.153	0.508*

*For non-normal distributed data, Spearman-Rho correlations were calculated

^a^Number line performance of children younger than 12 years is based on the percentage error between indicated and correct location of an Arabic digit, the solution of an addition or subtraction problem, and estimated number of dots on a paper-and-pencil number line task 0–100. For children older than 12 number line performance is measured by a computerized number line task 0–100 including Arabic digits and the solution of addition or subtraction problems

^b^Percentage of correctly solved addition and subtraction problems, respectively, in the number line task

^c^Mean IQ of children younger than 12 years is based on the mean of the subtests similarities, block design, digit span, picture concepts, vocabulary, and arithmetic of the WISC-IV and of children older than 12 years is based on the mean of the subtests similarities, block design, digit span and matrix reasoning of the WISC-IV

^d^Working memory capacity is based on the number of memories and correctly repeated span of the CORSI-Block Suppression test

^e^Mathematical performance of children younger than 12 years is based on the total reached percentile rank (PR) in the ZAREKI-R test battery and of children older than 12 years on the total score of the BASIS-Math test battery

^f^Arithmetical fluency of children younger than 12 years is based on the mean of correctly solved addition, subtraction, multiplication, and division problems within 2 min each of the HRT test

^g^Reading skills of children younger than 12 years is based on the subtest reading of the BUEGA test battery and of children older than 12 years on the correctly read words and pseudowords of the SLRT-II

^h^Magnitude comparison skills of children older than 12 years is based on the mean t-values of the subtest quantity of the KFT4-8+R test battery

Statistically significant values are marked bold

Differences between DD and CC were evaluated by multivariate analysis of variance (MANOVA) including all brain volumes of subcortical or cortical areas as dependent variable and group as independent variable (DD or CC). In addition, total intracranial volume, age and mean IQ were added as nuisance factors to control for confounding effects of total intracranial volume and group effects due to age or IQ since DD and CC differed significantly in these variables.

## Results

### Behavioral findings

As expected, children with DD performed worse in tests measuring numerical competence in a wide range of skills from basic numerical abilities (magnitude comparison, counting, transcoding between magnitudes and Arabic digits etc.) to higher mathematical operations (arithmetic, word problems, fractions etc.), even when controlling for age (please see Table 1). Intelligence was in the normal range for all children, however, lower in children with DD. IQ measures are known to be not fully independent from measures of math ability, and the present sample therefore reflects the cognitive pattern typically observed in DD. Moreover, it is important to note that our results did not change if IQ values were controlled in the analysis (see supplementary materials “Control for Age and Intelligence”).

Regarding math anxiety, our findings demonstrate that domain-specific anxiety was present in children with and without DD, however, children with DD showed higher scores of math anxiety compared to children without math problems (see Table 1). This difference in math anxiety was also observed when controlling for age, since the CC were slightly younger than the DD children were.

Pearson’s correlation between math anxiety and demographic and behavioral measurements revealed that children with increased math anxiety performed worse in arithmetic tasks to be solved with and without time pressure (please see Table 2). However, math anxiety was not significantly related to the outcome of extensive mathematical test batteries bearing on a wide range numerical skills such as non-symbolic and symbolic number processing, counting abilities, visuo-spatial number representation, or higher mathematical skills requiring the understanding of calculation procedures (please see ZAREKI-R and BASIS MATH in Table 2). No significant relationships between math anxiety and general factors, such as age, intelligence, or working memory were found. Additionally, no association between math anxiety and domain-remote skills, as reading was evident.

Regarding gender differences, our results indicated that girls and boys performed at equal cognitive levels (addition, subtraction, number line deviation, mathematical performance, magnitude comparison, intelligence, working memory, or reading) and experienced math anxiety equally often.

### Brain findings

**Fig. 1:**
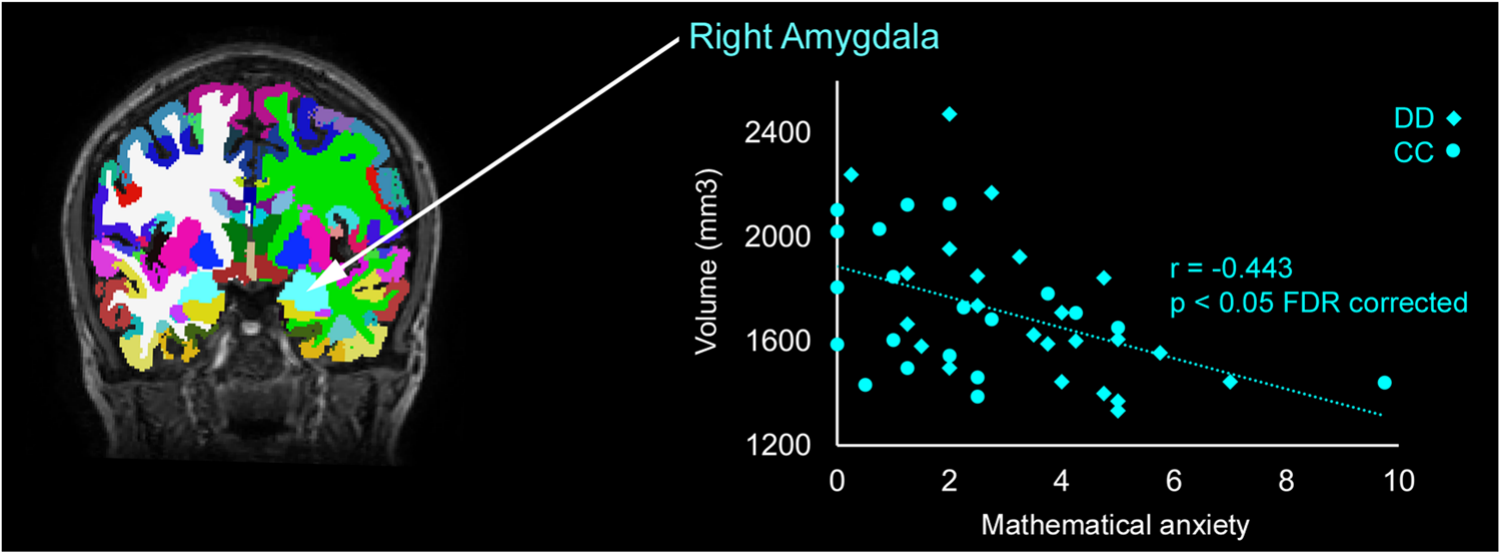
Reduced amygdala volume with increased math anxiety. Right amygdala volume is significantly smaller in children with developmental dyscalculia (DD, diamonds) and control children (CC, circles) with increasing math anxiety (0 = no math anxiety, 10 = very strong math anxiety)

Math anxiety was significantly related to the volume of the right amygdala (*r* = −0.443, *p* < 0.005, FDR-corrected *p* < 0.05), the anterior corpus callosum (*r* = −0.334, *p* < 0.05, FDR-corrected *p* = 0.188), the right inferior frontal sulcus (*r* = 0.321, *p* < 0.05, FDR-corrected *p* = 0.920), and the pericallosal sulcus (*r* = 0.388, *p* < 0.05, FDR-corrected *p* = 0.952) (see Table S1 for subcortical and central volumes and Table S2 for cortical volumes). Only the volume of the right amygdala survived the FDR-correction, indicating that higher levels of mathematical anxiety are related to smaller right amygdala volumes (see Fig. 1).

Further statistical analyses, taking differences between children with and without DD into account by either separate data analyses for children with or without DD or by including age, intelligence, addition, and subtraction as nuisance factors into statistical analyses supported the observation that the right amygdala volume is negatively correlated with math anxiety (please see supplementary materials “Separated for Groups” and “Control for Age and Intelligence”).

## Discussion

Mathematical anxiety is a widespread problem. As many as a quarter of the children are believed to be affected my math anxiety, but it is not always recognized or understood. The present findings demonstrate for the first time that mathematical anxiety is even related to changes brain structure. These findings have significant implications for diagnostics and treatment as they ultimately proof that math anxiety in children is real.

The most significant result of the present study represents the neuronal relations of math anxiety. Our findings demonstrated for the first time that math anxiety in children is associated with alterations in brain volume. Notably, structural changes were present in the amygdala, which represents the key area in our brain for negative emotional processing such as fear, stress and anxiety. Putting these results into the context of those from existing studies is challenging because no previous studies examined the relation between math anxiety and brain structure. However, some insights into the effects of negative emotions associated with mathematics on brain function have been gained in the last years. To our knowledge, only two studies have examined children^14,15^. Both reported stronger activation of the right amygdala in 7 to 9 year old children with high math anxiety. Moreover, functional connectivity of the right amygdala with other brain areas responsible for the regulation of negative emotions or mathematical processing was also stronger in math anxious children. In line with reported findings of brain function, it is impressive that math anxiety in children can be traced to altered brain function, brain connectivity and finally, as demonstrated in the present study, even to the brain structure that has been consistently implicated in specific and generalized anxieties, namely the amygdala.

There is ample evidence for hemispheric lateralization in emotional processing, but outcome of brain asymmetry in the amygdala remains to be clarified^49^. Present findings provide further evidence that the amygdala is essential for negative emotional processing and that there are hemisphere-specific processing differences in the amygdala. In particular, our findings support the involvement of the right amygdala in anxiety and negative emotions, as repeatedly reported in animal models and in humans (e.g.^50^). However, the specific roles of the left and right amygdala in emotional processing are still inconclusive and need to be clarified in future studies. In contrast to the functional laterality of brain areas during anxiety and stress, which have been studied relatively widely, structural asymmetries are still poorly documented (reviewed by 24). Overall, and consistent with present findings, the effects of anxiety and stress on brain structure were mostly reported in the amygdala among other regions^24^. Although findings regarding the volume or laterality of the amygdala are mixed, a multitude of studies reported that the amygdala is both smaller and more reactive to emotional stimuli in humans with anxiety disorders (reviewed by ref. ^51^). For instance, a negative relation between amygdala volume and anxiety has been reported in pediatric clinical anxiety disorder^52^, in subclinical state and trait anxiety measures in adults^53^, and in anxiety scores of pediatric populations with stress related disorders, such as post-traumatic-stress-disorder, adrenal hyperplasia, or Cushing syndrome (reviewed by ref. ^24^). This tendency towards smaller amygdala volume has been suggested to arise from stress-induced neural plasticity and seems to be inversely related to volumes of anxiety modulating cortical brain structures, like the inhibitory control of prefrontal cortices, which show larger volumes in anxiety^54^. The mechanism that leads to volume changes of brain areas after experience of acute or chronic stressors is probably multifactorial. Consideration of the age at which adverse events occurred and the age of assessment has been proposed to explain the seemingly discrepant findings across studies. There is also consistent evidence that initially the amygdala volume increases following a stressor and is accompanied by hyperactivity. Chronic hyperactivity of the amygdala then results in cellular atrophy and/or death and smaller amygdala volumes measured by MRI (reviewed by ref. ^51^). Such a trajectory would explain why children and adolescents who experienced years of math anxiety show decreased amygdala volume. It is important to note that we have to assume that these observed detrimental effects of math anxiety remain into adulthood if not addressed, since studies in adults have still found decreased amygdala volumes in individuals with a history of stressful childhood (reviewed by ref. ^51^. However, it has to be noted that the present study is not longitudinal and does not allow for any causal directionality of effects.

Finally, we also looked at group differences in brain volume, but no differences in volumes for subcortical (Wilks’ *λ* = 0.006, *F*(2, 38) = 4.313, *p* = 0.367) or cortical areas (Wilks’ *λ* = 0.004, *F*(2, 38) = 6.300, *p* = 0.307) were found between children with and without DD. Therefore, smaller brain volumes in DD cannot explain detected volume differences in the amygdala with increased math anxiety.

On the behavioral level, our findings corroborate that mathematical anxiety is present in children with and without DD, however, children with DD showed higher scores of math anxiety compared to children without math problems. These findings are consistent with results from previous studies reporting that math anxiety seems to be associated with math learning problems in clinical and even nonclinical samples of low math achievers^9^. Hence, our findings underscore the importance of considering emotional factors when characterizing children with specific math learning problems.

Independent of dyscalculia, children with increased math anxiety performed worse in arithmetic and were less fluent in solving arithmetic problems. However, math anxiety was not significantly related to the outcome of mathematical test batteries including a wide range of numerical abilities like non-symbolic and symbolic number processing, counting abilities, visuo-spatial number representation, or higher mathematical skills requiring the understanding of calculation procedures. In general, a large body of evidence confirms that math anxiety severely interferes with math learning and performance, both because math anxious people are more likely to avoid mathematical activities and because math anxiety usurps working memory resources (reviewed by ref. ^3^). Moreover, our findings corroborate the recent assumption by Dowker et al.^3^ that “it would appear likely that if anxiety affects working memory, it would have a particularly strong effect on arithmetic, as working memory has been found in many studies to be strongly associated with arithmetical performance…”. In this line, present findings show most prominent negative effects of math anxiety on arithmetic and arithmetical fluency.

No association between math anxiety and general factors, like age, intelligence, or working memory were found. In addition, no relation between math anxiety and domain-remote skills, such as reading was evident. In general, studies suggest that math anxiety increases with age, although studies differ with regard to the age at which specific math anxiety is assumed to emerge, and math anxiety tends to deteriorate with further development. In addition, the reasons why math anxiety tends to increase as children get older are unclear and seem to depend on the tests that have been used to evaluate math anxiety. General developmental effects like an increase of general anxiety or working memory with age and even cultural differences have been discussed^3^. Importantly, the present study rather suggests that math anxiety is already present in 8-year old children, which is consistent with reports suggesting that math anxiety can be detected in the earliest stages of formal math learning in school^55,56^. In terms of domain-general abilities, it has been suggested that poor intellectual conditions (e.g., poor abstract thinking or poor visuospatial skills) may contribute to the development of math anxiety (reviewed by ref. ^19^). All Children in the present study performed within the normal IQ-range and exhibited no other domain-general problems. Hence, the present findings suggest that math anxiety is unrelated to domain-general abilities in typically achieving children and children with DD.

Regarding differences between boys and girls, no disparities were evident at cognitive levels or experienced math anxiety. This is a promising result regarding the widely and ever since discussed stereotype that females are expected to be worse in math related topics and that females experience more math anxiety (e.g., discussed in science already in 1987 by ref. ^57^). Our findings are consistent with research indicating that countries providing equal education for females and males show little or no gender differences in mathematical performance^28,58^. However, females still tend to rate their mathematical competence lower and their anxiety levels higher than males (e.g.^59,60^). The reason why no gender differences in math anxiety were noticeable might be due to increasing evidence that such gender differences only develop at adolescence as consequence of societal exposure to gender stereotypes (e.g.^61^), or female teachers who experience math anxiety themselves^8^. In contrast, several studies report that younger children in primary school do not exhibit gender differences in math anxiety (e.g.^60,62^). Our findings are consistent with these reports since children in our study were mostly in primary school or just at the beginning of adolescence. In terms of gender differences, the present findings might also reflect positive developments in social attitudes regarding math and gender equality in education.

### Math anxiety: a serious problem

Mathematical anxiety has attracted the interest of the research community only recently, but recent results emphasize that math anxiety already exists at the beginning of formal schooling, interferes negatively with mathematical performance, especially in tasks requiring arithmetical knowledge, and has adverse long-term consequences for school and professional success. Furthermore, recent brain imaging findings provide evidence that the functional brain correlates of math anxiety are similar to those observed in general or in other types of specific anxieties, and present findings demonstrate for the first time volume changes in math anxious children. Hence, considering the reported high prevalence rates there seems to be no doubt that mathematical anxiety represents a serious problem for overall educational outcomes in society.

However, regarding the relationship between math anxiety and structural brain changes, the question of causation is difficult to pinpoint: Does math anxiety lead to a volume decrease of the amygdala, or does a preexisting smaller amygdala volume makes one more anxious? Although it is impossible to draw clear conclusions about causality from the present findings, directionality between negative experiences and amygdala structure and function has been confirmed with animal models of stress, which have consistently described changes in the amygdala following stress exposure (reviewed by ref. ^63^). Accordingly, we assume that the feelings of tension, stress, and frustration due to anxiety about failure in mathematics and anxiety in math-related situations can lead to a shrinkage of the right amygdala in children with and without DD. However, this should be further investigated in a longitudinal study.

It has also to be considered that we did not assess general anxiety or test anxiety in the present study. Therefore, reduced amygdala volume could be related to anxiety in general, rather than math anxiety in particular. However, it is important to mention that although math anxiety seems to be related to other measures of anxiety, it cannot be reduced purely to these more general measures. Kohn and colleagues^28^ investigated the relations of math anxiety with the identical math anxiety interview as in the present study and found that the association of math anxiety and math performance is not explicable by general cognitive abilities and not by general anxiety or school- and test anxiety.

Although changes in brain structure due to negative experiences during childhood seem to persist into adulthood, specific interventions might have the potential to reverse detrimental effects of mathematical anxiety. Remarkably, studies that have examined the effects of math interventions on the emotional experience of performing math or math related situations could demonstrate that even relatively short tutoring of approximately 2 month induces a reduction in mathematical anxiety^15,64^. Moreover, math tutoring normalized hyperactivity and functional connectivity of the amygdala in children with high math anxiety to the level of their low math anxious peers^15^. These positive effects give rise to the hope that interventions based on exposure to mathematical material would also be able to induce a normalization of amygdala volumes in math anxious individuals.

To conclude, present findings add strong evidence that mathematical anxiety is present at the beginning of formal schooling and not only hinders children in developing mathematical abilities, but it is also associated with altered brain structure in negative emotional circuits implicated in other types of anxiety. This growing knowledge underscores the important role of emotional factors in mathematical cognition and encourages educators and researchers alike to consider math anxiety, especially in children with DD, since these children are particularly prone to develop math anxiety.

## Supplementary information

Table S1
Table S2
Supplementary Data
