# Supplementary material for: Neurostructural correlate of math anxiety in the brain of children
Source: Transl Psychiatry. 2018 Dec 10;8:273. doi: 10.1038/s41398-018-0320-6 (PMC6288142; doi:10.1038/s41398-018-0320-6)
Supplement: Supplementary file 1 — Table S1 [file 41398_2018_320_MOESM1_ESM.docx]

Table S1: 26 Subcortical Volumes

| ***Subcortical volume*** ^a^ | | ***Normality*** ^b^ | ***Partial correlation with math anxiety*** ^c^ | | |
| --- | --- | --- | --- | --- | --- |
|  |  | *p-value* | *Correlation coefficient r* | *p-value* | *FDR corrected* |
| Left | Cerebellum cortex | .200 | .079 | .633 | .918 |
|  | Thalamus | .200 | .026 | .877 | .918 |
|  | Caudate | .200 | .017 | .918 | .918 |
|  | Putamen | .200 | .051 | .758 | .918 |
|  | Pallidum | .200 | .036 | .827 | .918 |
|  | Hippocampus | .182 | .034 | .835 | .918 |
|  | Amygdala | .200 | -.209 | .203 | .918 |
|  | Accumbens area | **.020** | -.142 | .388 | bootstrap |
|  | Ventral diencephalon | .200 | -.149 | .366 | .918 |
| Right | Cerebellum cortex | .200 | .027 | .870 | .870 |
|  | Thalamus | .200 | .161 | .328 | .870 |
|  | Caudate | .200 | .042 | .797 | .870 |
|  | Putamen | .165 | -.030 | .857 | .870 |
|  | Pallidum | .200 | -.133 | .418 | .870 |
|  | Hippocampus | .200 | .088 | .595 | .870 |
|  | **Amygdala** | .200 | **-.443** | **.005** | **.042** |
|  | Accumbens area | .200 | -.163 | .322 | .870 |
|  | Ventral diencephalon | .200 | -.082 | .620 | .870 |
| Central | Optic chiasm | .200 | .131 | .427 | .534 |
|  | Posterior corpus callosum | .200 | -.165 | .316 | .534 |
|  | Middle posterior corpus callosum | **.002** | -.268 | .099 | bootstrap |
|  | Central corpus callosum | **.011** | -.133 | .419 | bootstrap |
|  | Middle anterior corpus callosum | **.011** | -.175 | .287 | bootstrap |
|  | **Anterior corpus callosum** | .200 | **-.334** | **.038** | .188 |
|  | Brain stem | .200 | -.059 | .722 | .722 |
|  | Cortico spinal fluid | .200 | .144 | .382 | .534 |

^a^ Subcortical volume labels by freesurfer.

^b^ Normal distribution was tested by Kolmogorov-Smirnov test. For volumes violating the assumption of normality (indicated by significant p-values), bootstrapping was applied.

^c^ Partial correlation between mathematical anxiety and different volumes were controlled for total intracranial volume, addition, and subtraction performance. Partial correlation coefficient r, p-value, and FDR corrected p-value for multiple comparisons are listed.
