# Supplementary material for: Neurostructural correlate of math anxiety in the brain of children
Source: Transl Psychiatry. 2018 Dec 10;8:273. doi: 10.1038/s41398-018-0320-6 (PMC6288142; doi:10.1038/s41398-018-0320-6)
Supplement: Supplementary file 2 — Table S2 [file 41398_2018_320_MOESM2_ESM.docx]

Table S2: 148 Cortical Volumes

| ***Cortical volume*** ^a^ | | ***Normality*** ^b^ | ***Partial correlation with math anxiety*** ^c^ | | |
| --- | --- | --- | --- | --- | --- |
|  |  | *p-value* | *Correlation coefficient r* | *p-value* | *FDR corrected* |
| Right | Fronto-marginal gyrus and sulcus | .200 | .094 | .568 | .920 |
|  | Inferior occipital gyrus and sulcus | .200 | -.262 | .107 | .920 |
|  | Paracentral lobule and sulcus | .200 | -.040 | .808 | .959 |
|  | Subcentral gyrus and sulci | .200 | .143 | .385 | .920 |
|  | Transverse frontopolar gyri and sulci | .200 | -.109 | .507 | .920 |
|  | Anterior cingulate gyrus and sulcus | .200 | .015 | .928 | .962 |
|  | Middle-anterior cingulate gyrus and sulcus | .200 | .143 | .386 | .920 |
|  | Middle-posterior cingulate gyrus and sulcus | .200 | .225 | .168 | .920 |
|  | Posterior-dorsal cingulate gyrus | .200 | .095 | .565 | .920 |
|  | Posterior-ventral cingulate gyrus | .200 | .093 | .574 | .920 |
|  | Cuneus | .068 | .039 | .816 | .959 |
|  | Opercular part of the inferior frontal gyrus | .200 | .116 | .481 | .920 |
|  | Orbital part of the inferior frontal gyrus | .200 | .073 | .658 | .920 |
|  | Triangular part of the inferior frontal gyrus | .200 | .089 | .589 | .920 |
|  | Middle frontal gyrus | .200 | -.027 | .869 | .962 |
|  | Superior frontal gyrus | .200 | .147 | .372 | .920 |
|  | Long insular gyrus and central sulcus of the insula | .186 | -.020 | .906 | .962 |
|  | Short insular gyri | .200 | .095 | .564 | .920 |
|  | Middle occipital gyrus | .200 | -.288 | .076 | .920 |
|  | Superior occipital gyrus | .200 | -.101 | .542 | .920 |
|  | Lateral occipito-temporal gyrus | .128 | .183 | .265 | .920 |
|  | Lingual gyrus | .190 | -.095 | .567 | .920 |
|  | Parahippocampal gyrus | .148 | .104 | .530 | .920 |
|  | Orbital gyri | .200 | .202 | .219 | .920 |
|  | Angular gyrus | .200 | -.124 | .451 | .920 |
|  | Supramarginal gyrus | .200 | .088 | .592 | .920 |
|  | Superior parital lobe | .200 | -.290 | .073 | .920 |
|  | Postcentral gyrus | .200 | -.155 | .346 | .920 |
|  | Precentral gyrus | **.004** | -.139 | .399 | bootstrap |
|  | Precuneus | .200 | .095 | .564 | .920 |
|  | Straight gyrus | .200 | .008 | .963 | .978 |
|  | Subcallosal gyrus | .200 | -.080 | .626 | .920 |
|  | Anterior transverse temporal gyrus | .200 | .255 | .117 | .920 |
|  | Lateral aspect of the superior temporal gyrus | .200 | .054 | .743 | .959 |
|  | Planum polare of the superior temporal gyrus | .200 | .022 | .892 | .962 |
|  | Planum temporale | .200 | .113 | .493 | .920 |
|  | Inferior temporal gyrus | .200 | .193 | .239 | .920 |
|  | Middle temporal gyrus | .200 | .030 | .855 | .962 |
|  | Horizontal ramus of the anterior segment of the lateral sulcus | **.033** | .096 | .561 | bootstrap |
|  | Vertical ramus of the anterior segment of the lateral sulcus | **.010** | .171 | .298 | bootstrap |
|  | Posterior ramus of the lateral sulcus | .200 | -.015 | .926 | .962 |
|  | Occipital pole | .159 | -.019 | .907 | .962 |
|  | Temporal pole | .200 | -.092 | .576 | .920 |
|  | Calcarine sulcus | .200 | .165 | .315 | .920 |
|  | Central sulcus | **.043** | -.027 | .870 | bootstrap |
|  | Marginal branch of the cingulate sulcus | .075 | .042 | .802 | .959 |
|  | Anterior segment of the circular sulcus of the insula | .200 | .090 | .585 | .920 |
|  | Inferior segment of the circular sulcus of the insula | .188 | .298 | .065 | .920 |
|  | Superior segment of the circular sulcus of the insula | .200 | .045 | .783 | .959 |
|  | Anterior transverse collateral sulcus | .200 | .232 | .156 | .920 |
|  | Posterior transverse collateral sulcus | .098 | -.269 | .097 | .920 |
|  | **Inferior frontal sulcus** | .200 | **.321** | **.046** | .920 |
|  | Middle frontal sulcus | .200 | -.148 | .368 | .920 |
|  | Superior frontal sulcus | .200 | -.116 | .481 | .920 |
|  | Sulcus intermedius primus of Jensen | **.000** | -.008 | .961 | bootstrap |
|  | Intraprarietal sulcus and transverse parital sulci | .200 | .014 | .933 | .962 |
|  | Middle occipital sulcus and lunatus sulcus | .200 | -.003 | .983 | .983 |
|  | Superior occipital sulcus and transverse occipital sulcus | .200 | -.046 | .782 | .959 |
|  | Anterior occipital sulcus and preoccipital notch | .200 | -.082 | .620 | .920 |
|  | Lateral occipito-temporal sulcus | .200 | -.237 | .147 | .920 |
|  | Collateral sulcus and lingual sulcus | .172 | -.046 | .779 | .959 |
|  | Lateral orbital sulcus | .200 | .061 | .713 | .959 |
|  | Olfactory sulcus | .200 | -.125 | .449 | .920 |
|  | Orbital sulci | .155 | .132 | .424 | .920 |
|  | Parieto-occipital sulcus | .200 | .057 | .729 | .959 |
|  | Sulcus of corpus callosum | .200 | .088 | .595 | .920 |
|  | Postcentral sulcus | .200 | -.229 | .160 | .920 |
|  | Inferior part of the precentral sulcus | .200 | .092 | .576 | .920 |
|  | Superior part of the precentral sulcus | **.005** | -.087 | .596 | bootstrap |
|  | Suborbital sulcus | .200 | -.107 | .517 | .920 |
|  | Subparietal sulcus | **.036** | -.184 | .263 | bootstrap |
|  | Inferior temporal sulcus | .200 | -.073 | .659 | .920 |
|  | Superior temporal sulcus | .200 | .087 | .599 | .920 |
|  | Transverse temporal sulcus | .200 | .079 | .633 | .920 |
| Left | Fronto-marginal gyrus and sulcus | .200 | -.092 | .577 | .976 |
|  | Inferior occipital gyrus and sulcus | .200 | .112 | .496 | .976 |
|  | Paracentral lobule and sulcus | .200 | .037 | .822 | .976 |
|  | Subcentral gyrus and sulci | .200 | -.007 | .968 | .998 |
|  | Transverse frontopolar gyri and sulci | .200 | .003 | .958 | .998 |
|  | Anterior cingulate gyrus and sulcus | .200 | -.064 | .699 | .976 |
|  | Middle-anterior cingulate gyrus and sulcus | .200 | .058 | .725 | .976 |
|  | Middle-posterior cingulate gyrus and sulcus | .200 | .204 | .213 | .952 |
|  | Posterior-dorsal cingulate gyrus | .200 | -.047 | .778 | .976 |
|  | Posterior-ventral cingulate gyrus | .129 | .146 | .374 | .976 |
|  | Cuneus | .200 | -.077 | .642 | .976 |
|  | Opercular part of the inferior frontal gyrus | .200 | .050 | .763 | .976 |
|  | Orbital part of the inferior frontal gyrus | .200 | .218 | .183 | .952 |
|  | Triangular part of the inferior frontal gyrus | .200 | .024 | .882 | .987 |
|  | Middle frontal gyrus | .200 | -.131 | .426 | .976 |
|  | Superior frontal gyrus | .200 | .014 | .933 | .998 |
|  | Long insular gyrus and central sulcus of the insula | .099 | .213 | .192 | .952 |
|  | Short insular gyri | .200 | .066 | .690 | .976 |
|  | Middle occipital gyrus | .200 | -.086 | .601 | .976 |
|  | Superior occipital gyrus | .200 | .053 | .748 | .976 |
|  | Lateral occipito-temporal gyrus | .060 | -.034 | .839 | .976 |
|  | Lingual gyrus | .172 | -.307 | .058 | .952 |
|  | Parahippocampal gyrus | .200 | -.169 | .303 | .952 |
|  | Orbital gyri | .200 | .134 | .415 | .976 |
|  | Angular gyrus | .200 | -.111 | .500 | .976 |
|  | Supramarginal gyrus | **.021** | .085 | .606 | bootstrap |
|  | Superior parital lobe | .200 | -.192 | .241 | .952 |
|  | Postcentral gyrus | .200 | -.190 | .246 | .952 |
|  | Precentral gyrus | .200 | -.155 | .345 | .976 |
|  | Precuneus | .200 | -.050 | .763 | .976 |
|  | Straight gyrus | .200 | -.105 | .523 | .976 |
|  | Subcallosal gyrus | .200 | -.087 | .600 | .976 |
|  | Anterior transverse temporal gyrus | .200 | .270 | .096 | .952 |
|  | Lateral aspect of the superior temporal gyrus | .200 | .075 | .651 | .976 |
|  | Planum polare of the superior temporal gyrus | .200 | -.069 | .675 | .976 |
|  | Planum temporale | .200 | -.239 | .144 | .952 |
|  | Inferior temporal gyrus | .200 | .086 | .603 | .976 |
|  | Middle temporal gyrus | .200 | -.008 | .960 | .998 |
|  | Horizontal ramus of the anterior segment of the lateral sulcus | **.025** | -.099 | .549 | bootstrap |
|  | Vertical ramus of the anterior segment of the lateral sulcus | .200 | .202 | .217 | .952 |
|  | Posterior ramus of the lateral sulcus | .200 | .145 | .379 | .976 |
|  | Occipital pole | .200 | .100 | .546 | .976 |
|  | Temporal pole | .200 | .033 | .842 | .976 |
|  | Calcarine sulcus | .200 | .105 | .526 | .976 |
|  | Central sulcus | .200 | -.129 | .434 | .976 |
|  | Marginal branch of the cingulate sulcus | .200 | .101 | .539 | .976 |
|  | Anterior segment of the circular sulcus of the insula | .061 | .211 | .198 | .952 |
|  | Inferior segment of the circular sulcus of the insula | .200 | -.003 | .988 | .998 |
|  | Superior segment of the circular sulcus of the insula | .200 | .239 | .143 | .952 |
|  | Anterior transverse collateral sulcus | .200 | -.056 | .736 | .976 |
|  | Posterior transverse collateral sulcus | .200 | -.046 | .779 | .976 |
|  | Inferior frontal sulcus | **.001** | .087 | .596 | bootstrap |
|  | Middle frontal sulcus | .078 | .218 | .182 | .952 |
|  | Superior frontal sulcus | .200 | .182 | .267 | .952 |
|  | Sulcus intermedius primus of Jensen | **.018** | .025 | .880 | bootstrap |
|  | Intraprarietal sulcus and transverse parital sulci | **.036** | -.117 | .476 | bootstrap |
|  | Middle occipital sulcus and lunatus sulcus | .200 | -.250 | .125 | .952 |
|  | Superior occipital sulcus and transverse occipital sulcus | .200 | .208 | .204 | .952 |
|  | Anterior occipital sulcus and preoccipital notch | .200 | -.103 | .531 | .976 |
|  | Lateral occipito-temporal sulcus | .200 | -.074 | .654 | .976 |
|  | Collateral sulcus and lingual sulcus | .200 | -.216 | .186 | .952 |
|  | Lateral orbital sulcus | **.044** | -.024 | .883 | bootstrap |
|  | Olfactory sulcus | **.035** | .003 | .987 | bootstrap |
|  | Orbital sulci | .200 | .032 | .849 | .976 |
|  | Parieto-occipital sulcus | .200 | .179 | .276 | .952 |
|  | **Sulcus of corpus callosum** | .200 | **.388** | **.015** | .952 |
|  | Postcentral sulcus | .200 | -.201 | .220 | .952 |
|  | Inferior part of the precentral sulcus | .200 | -.030 | .858 | .976 |
|  | Superior part of the precentral sulcus | .200 | -.041 | .806 | .976 |
|  | Suborbital sulcus | **.033** | -.041 | .805 | bootstrap |
|  | Subparietal sulcus | .200 | .018 | .915 | .998 |
|  | Inferior temporal sulcus | .200 | -.173 | .291 | .952 |
|  | Superior temporal sulcus | .200 | .000 | .998 | .998 |
|  | Transverse temporal sulcus | .200 | .116 | .481 | .976 |

^a^ Cortical volume labels by freesurfer.

^b^ Normal distribution was tested by Kolmogorov-Smirnov test. For volumes violating the assumption of normality (indicated by significant p-values), bootstrapping was applied.

^c^ Partial correlation between mathematical anxiety and different volumes were controlled for total intracranial volume, addition, and subtraction performance. Partial correlation coefficient r, p-value, and FDR corrected p-value for multiple comparisons are listed.
