# Supplementary material for: Neurostructural correlate of math anxiety in the brain of children
Source: Transl Psychiatry. 2018 Dec 10;8:273. doi: 10.1038/s41398-018-0320-6 (PMC6288142; doi:10.1038/s41398-018-0320-6)
Supplement: Supplementary file 3 — Supplementary Data [file 41398_2018_320_MOESM3_ESM.docx]

**Supplementary Material**

Separated Groups

Partial correlation was performed for children with and without DD separately. Correlation between math anxiety and subcortical, central, or cortical volumes, controlling for total intracranial volume, addition, and subtraction performance indicated for DD children a relation between math anxiety and the right amygdala volume (r = -0.563, p < 0.05) and a similar trend for control children (r = -0.451, p = 0.069). None of the other subcortical or central volumes showed a relation to math anxiety. Several cortical volumes were correlated with math anxiety in DD children: right Lingual gyrus (r = -0.562, p < 0.05), right postcentral gyrus (r = -0.507, p < 0.05), right inferior temporal sulcus (r = -0.562, p < 0.05), and left planum temporale (r = -0.475, p < 0.05). In control children following cortical volumes were related to math anxiety: right anterior transverse temporal gyrus (r = 0.506, p < 0.05), right inferior segment of the circular sulcus of the insula (r = 0.550, p < 0.05), right inferior frontal sulcus (r = 0.498, p < 0.05), left orbital part of the inferior frontal gyrus (r = 0.509, p < 0.05), left calcarine sulcus (r = 0.543, p < 0.05), left olfactory sulcus (r = 0.530, p < 0.05), and left sulcus of the corpus callosum (r = 0.489, p < 0.05). None of these correlations survived statistical correction for multiple comparison by FDR.

Control for Age and Intelligence

Second, partial correlation was performed including all children, but additionally controlling for parameters that differed significantly between children with and without DD (age and intelligence). Correlation between math anxiety and volumes, partialling out the effects of total intracranial volume, addition and subtraction performance, age and intelligence, showed, as well, comparable results - a significant relation between math anxiety and the right amygdala volume (r = -0.399, p < 0.05) and the anterior corpus callosum (r = -0.335, p < 0.05). Cortical volume of following areas correlated with math anxiety: right inferior occipital gyrus and sulcus (r = -0.369, p < 0.05), right middle occipital gyrus (r = -0.396, p < 0.05), left lingual gyrus (r = -0.358, p < 0.05), left collateral sulcus and lingual sulcus (r = -0.369, p < 0.05), and left sulcus of the corpus callosum (r = 0.345, p < 0.05). None of these correlations survived statistical correction for multiple comparison by FDR.
